# Efficacy of the Combined Administration of Systemic and Intra-Articular Tranexamic Acid in Total Hip Arthroplasty Secondary to Femoral Neck Fracture: A Retrospective Study

**DOI:** 10.1155/2020/9130462

**Published:** 2020-04-14

**Authors:** Joseph Maalouly, Antonios Tawk, Rami Ayoubi, Georges Katoul Al Rahbani, Aida Metri, Elias Saidy, Gerard El-Hajj, Alexandre Nehme

**Affiliations:** ^1^Department of Orthopedic Surgery and Traumatology, Saint George Hospital University Medical Center, Balamand University, P.O. Box 166378, Achrafieh, Beirut 1100 2807, Lebanon; ^2^Faculty of Medicine and Medical Sciences, University of Balamand, Aschrafieh, Beirut, Lebanon; ^3^Department of Medical Imaging and Radiology, Saint George Hospital University Medical Center, Balamand University, P.O. Box 166378, Achrafieh, Beirut 1100 2807, Lebanon

## Abstract

**Background:**

Total hip arthroplasty (THA) is associated with substantial blood loss in the postoperative course. Tranexamic acid (TXA) is a potent antifibrinolytic agent, routinely administered by intravenous (IV) and topical (intra-articular, IA) route, which can possibly interrupt the cascade of events due to hemostatic irregularities close to the source of bleeding. However, scientific evidence of combined administration of TXA in THA secondary to a femoral neck fracture is still meagre. The present study aims to compare the patients who were administered combined IV and topical TXA with a control group in terms of blood loss, transfusion rate, and incidence of deep vein thrombosis (DVT) and thromboembolism (TE). *Patients and Methods*. 195 patients with femoral neck fracture underwent THA and were placed into two groups: (1) IV and IA TXA group which had 58 patients and (2) no TXA control group which had 137 patients. In the TXA group, 1 g IV TXA was administered 30 minutes before incision, and 1 g IA TXA was administered intraoperatively after fascia closure. No drains were placed, and soft spica was applied to the hip.

**Results:**

Combined usage of IV and IA TXA showed better results when compared to the control group in terms of blood transfusion rate (31%) and hemoglobin drop (28%). No cases of DVT or TE were noted among the two study groups.

**Conclusion:**

Combined use of IV and IA TXA provided significantly better results compared to no TXA use with respect to all variables related to postoperative blood loss in THA. Moreover, TXA use is safe in terms of incidence of symptomatic DVT and TE.

## 1. Introduction

Large amounts of perioperative blood loss and large transfusion rates are associated with total joint replacement surgeries. Total hip arthroplasty (THA) patients are transfused at rates of 16–37% [1]. Femoral neck fractures are pathological entities associated with high mortality and morbidity and are projected to reach more than 6 million cases in the next 30 years [2]. When compared to hemiarthroplasty, THA is associated with better functional outcome scores and decreased reported pain throughout the postoperative course. However, there is an increased risk in THA regarding blood loss, surgery time, and risk of dislocation [3]. The perioperative blood loss in THA may lead to hematomas formation and in certain cases to acute anemia, subsequently resulting in blood transfusions with their associated risks (cardiopulmonary events and transfusion reactions) and health care costs [4]. In addition, allogeneic blood transfusions can also raise the patient's risk of adverse immunological reaction, disease transmission, and postoperative infection [5]. Therefore, every operation stimulates the fibrinolytic process transiently [6]. There is no replacement, of course, for effective surgical technique and local hemostasis intraoperatively. Nevertheless, through various means such as hypotensive anesthesia [7], drain clamping [8], preoperative autologous blood donation [9], and the use of antifibrinolytic agents, a surgeon can reduce the risk of blood loss.

Tranexamic acid (TXA) is a potent antifibrinolytic agent, which may interrupt the cascade of events due to hemostatic irregularities close to the bleeding source. Tranexamic acid, a synthetic analogue of amino acid lysine, prevents fibrinolysis by competitively blocking plasminogen lysine-binding sites, resulting in reduced proteolytic activity on fibrin monomers and fibrinogen, resulting in clot stabilization. [10]. It reaches a concentration of 90%–100% in joints compared with its plasma concentration [11]. In addition, TXA concentrations of up to 10 mg/mL of blood do not influence platelet counts, coagulation times, or various coagulation factors in whole blood or citrated blood from healthy subjects [12].

However, isolated case reports of thrombus formation have been reported, leading to concerns about the risk of thromboembolic complications in a patient population that is already at high risk for deep vein thrombosis (DVT) and pulmonary embolism (PE). This prevented widespread acceptance of IV antifibrinolytics in full joint replacement operations [13, 14].

Furthermore, multiple studies [15–17] including a meta-analysis [18] showed the potential of topical (IA) TXA to be equivalent or even better than intravenous (IV) TXA. In addition, a recent study by Lin et al. [19] found that the efficacy of combined TXA administration is higher than topical use alone. Thus, it is imperative to say that a combined administration of TXA may prove to be more effective compared with topical or intravenous TXA use alone.

The present study was therefore conducted to compare the efficacy of the combined use of IV and IA TXA with that of no use of TXA in terms of total blood loss and allogeneic transfusion rate and to assess the safety profile of each regimen in terms of DVT and TE incidence. As the additional use of topical TXA increases the probability of enhanced antifibrinolytic activity, we hypothesized that there will be a significant difference between the group with the combined use of IV and IA TXA and the group without TXA use in terms of blood loss, allogeneic blood transfusion rate, and hemoglobin (Hb) drop.

## 2. Materials and Methods

For this study, written informed consent was obtained from all the patients recruited.

A retrospective study of a single institution was conducted. All patients aged 50 to 100 diagnosed with femoral neck fracture and scheduled for primary arthroplasty by the senior author were eligible. The study excluded patients with history of renal impairment, cardiovascular disease (previous myocardial infarction, atrial fibrillation), or cerebrovascular disease (previous stroke or peripheral vascular surgery). Patients with history of thromboembolic disease, allergy to TXA, bleeding disorder, or receiving anticoagulant drug treatment were also excluded. Preoperative blood transfusions for patient preparation were not taken into account. Between January 2016 and September 2019, 195 patients (75 males and 120 females) were successfully recruited into two groups: (1) IV and IA TXA; and (2) no TXA.

30 minutes prior to incision, 1 g of IV TXA was given. All hip surgeries were operated with the standard posterolateral approach to the hip in order to decrease variability within the study, and noncemented prostheses were used. No drains were placed, and the closure of the wound was performed in a standard fashion in all cases. Soft spica was applied after dressing application. Intra-articular tranexamic acid 1 g in 20 mL NaCl was used after fascia closure in all surgeries. Postoperatively, all patients underwent a standard institution thromboembolic prophylaxis protocol. Pneumatic calf pumps were given immediately postoperatively until the patient started ambulating. As per the National Institute for Health and Care Excellence (NICE) recommendation, subcutaneous lovenox (enoxaparin sodium) 40 mg once daily (Sanofi, Paris, France) was given to all patients on the first postoperative day (POD) and continued until discharge from the hospital around 6 days postoperatively. Anticoagulation therapy was continued at home for a total of 35 days from the operation. All patients underwent inpatient postoperative physiotherapy with the aim of early mobilization. No screening for deep vein thrombosis was done in the postoperative period to detect asymptomatic occurrence. For symptomatic patients, evaluation with ultrasonography of the lower limb deep veins and CT scan of the chest (PE protocol) was done.

The primary outcomes of this study were transfusion incidences, postoperative drop in serum hemoglobin level while the secondary outcomes include duration of surgery, length of hospital stay, wound complications, and thromboembolic events within 30 days of surgery. Outcomes such as total blood loss, intraoperative blood, and hidden blood loss were not taken into account in this study.

At the institution where this study was performed, a serum hemoglobin level of less than 8.0 g/dl was considered the transfusion trigger. For patients presenting with anemic symptoms or any anemia-related organ dysfunctions, the transfusion trigger was less than 10.0 g/dl.

## 3. Statistical Analysis

Statistical analysis was carried out in consultation with the in-house biostatistician, using SPSS® 25.0 (IBM, Armonk, New York, United States). Statistical significance was defined as a *p* value of ≤0.05. Testing for normality was done with the Shapiro-Wilk test. We used Levene's test for equality of variances and Student's *t*-test for equality of means.

## 4. Results

**Figure 1:**
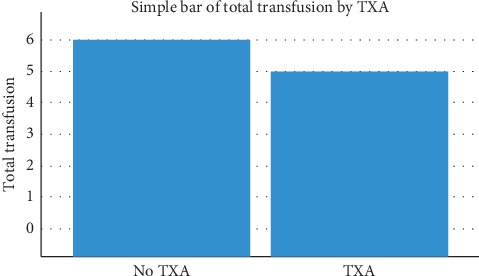
Superiority of the TXA regimen.

Our results show a decrease in the transfusion rate in the TXA group as seen in Figure 1. No statistically significant difference was found between the two studied groups in matter of age (Table 1) and BMI.  Table 2 shows that there is a significant negative Pearson correlation between TXA and blood transfusions (*p* < 0.05) and a positive Pearson correlation between TXA and hemoglobin levels on the first postoperative day. This is interpreted as tranexamic acid use decreases blood transfusions and leads to higher first day hemoglobin levels. Furthermore, a negative Pearson correlation is present between hemoglobin levels preoperatively and blood transfusions (*p* < 0.01), and a positive correlation between hemoglobin preoperatively and hemoglobin levels at day 1 and day 5 postoperatively (*p* < 0.01). This suggests that a higher preoperative hemoglobin reduces the need for transfusions and these patients have higher hemoglobin levels postoperatively. Levene's test was used for equality of variances and Student's *t*-test for equality of means as shown in Table 3. Our findings show no statistically significant difference in terms of preoperative hemoglobin levels and hemoglobin levels at day 5 postoperatively, while statistically significant difference between the two groups was noted in terms of day 1 postoperative hemoglobin levels and total amount of transfusions used intraoperatively and postoperatively. Since *p* < 0.05 is less than our chosen significance level *α*=0.05, we can reject the null hypothesis and conclude that statistically significant difference is present between the TXA group and the control group in terms of day 1 postoperative hemoglobin levels and total amount of transfusion.

## 5. Discussion

In the current study, the primary endpoint is the decrease in the hemoglobin drop and the decrease in transfusion rate seen with IV and IA administration of TXA in THA. The drop in hemoglobin (Hgb) was 28% lower in THA when TXA was used. This translated into a much lower risk of requiring a blood transfusion in the THA patients in which TXA was used. The patients with TXA required 31% less transfusion on average than the control group. The protocol used in this study consisted of 1 g IV tranexamic acid 30 minutes before incision and 1 g injection after fascia closure without placing any drains and with soft spica application looks to be effective and safe. There was no reported symptomatic thromboembolic event.

Currently, TXA is FDA approved only for tooth extraction in hemophilia patients. TXA distributes widely in the extracellular and intracellular compartments when given IV [20]. It spreads rapidly into the synovial fluid until the TXA concentration in the synovial fluid reaches the serum concentration [21]. Its biological half-life in the joint fluid is three hours, and glomerular filtration eliminates 90% within 24 hours [22, 23]. Compared to IV administration, intra-articular TXA advocates believe that the benefits include easy administration, maximum concentration at the bleeding site, and minimal systemic absorption. The objective of both delivery systems is to achieve a concentration of approximately 10 mg/mL [20].

Antifibrinolytics have been in use since the 1960s as a class of drugs [22]. TXA is an amino acid lysine analogue. It competitively inhibits the activation of plasminogen and the binding of plasmin to fibrin, inhibiting the degradation of fibrin [13]. Since it works by increasing the breakdown of fibrin when created, it is not necessarily a procoagulant but promotes coagulation already in progress. This makes TXA potentially suitable for use in reducing postoperative bleeding, where surgical hemostasis has been achieved and fibrinolytic activity needs to be suppressed to help maintain hemostasis without promoting the formation of venous thrombus.

In a meta-analysis of all operations, tranexamic acid has been shown to reduce the risk of transfusion by a third [23]. It was used successfully through an intravenous route in orthopedic surgery, with several studies showing significant reductions in bleeding and transfusion risk following THA [24]. Nevertheless, the possibility of thromboembolic complications following systemic administration remains a concern [13, 14].

Kagoma et al. [25] studied the impact of antifibrinolytics on blood transfusion reduction, showing a decrease in blood transfusion rate, after total knee and hip arthroplasties.

Rajesparan et al. reported less blood loss in the TXA group in a study of 73 patients undergoing THA [26]. Gill and Rosenstein found that the use of IV TXA in different doses significantly reduced intraoperative and total blood loss during the review of 13 randomized controlled trials [27].

Zhou et al. meta-analysis of 19 randomized control experiments using different doses of IV TXA in THA found marked reductions in total, intraoperative, postoperative, and secret blood loss in the TXA group compared to the control group [28].

No significant difference in safety between the different methods was noted after a comprehensive review of the literature [29]. For THA, Amin et al. suggested administering a dose of 15 mg/kg IV given 10 minutes before incision and 3 g in 100 mL of saline soak as defined intraoperatively by Melvin [30].

Based on the evidence in the literature, Hayes et al. recommend a regimen consisting of a 1 g IV dose of TXA to be administered before incision or tourniquet inflation with an additional 1 g IV dose given at closure [31].

Based on the current evidence-based medicine, clinicians and patients need to know the possible clinical benefits and risks of TXA, when deciding on their therapy management following THA. Regarding efficacy and safety, our study suggests that TXA should be indicated in patients undergoing THA secondary to femoral neck fracture. In view of our data, our protocol of 1 g IV tranexamic acid 30 minutes before incision and 1 g injection after fascia closure seems to be effective and safe. The dosing regimen appears to be one of the lowest reported dose in the literature with a good safety profile and efficiency.

## 6. Conclusion

After thorough review of the literature and according to our data, it is our recommendation that 1 g of TXA in IV be given 30 minutes prior to incision and 1 g TXA intra-articular after fascia closure without drains and with the application of a soft spica, as this is seen to be beneficial in terms of blood loss, blood transfusions, and postoperative hemoglobin drop. This seems important as it decreases hospital costs and risks to the patient from the transfusion and anemia. Our protocol appears safe and effective and may be the safest effective dosing regimen.

### 6.1. Limitations

This study has several limitations. First, the participants included in our study excluded high risk patients. These included patients with a history of cardiovascular disease, cerebrovascular disease, thromboembolic events, renal failure, allergy to TXA, bleeding diathesis, and those on anticoagulation therapy. Second, differences in surgical techniques and blood transfusion protocol are likely to have contributed to the differences observed among studies. Third, this is a retrospective study. Few studies reported the type of surgical hemostasis utilized; these techniques are known to reduce bleeding during surgery [31].

## Figures and Tables

**Table 1:** The demographics of the studied population in the current study.

Group statistics	Group	*N*	Mean	Std. deviation	Std. error mean
Pre-op Hgb	No TXA	137	12.133	1.9545	0.1670
	TXA	58	12.224	1.5226	0.1999

D1 PO Hgb	No TXA	137	10.560	1.3907	0.1188
	TXA	58	11.048	1.3806	0.1813

D5 PO Hgb	No TXA	137	10.251	1.0346	0.0884
	TXA	57	10.309	1.0863	0.1439

Total transfusion	No TXA	137	1.58	1.443	0.123
	TXA	58	1.10	1.209	0.159

Age	No TXA	137	70.42	16.478	1.408
	TXA	58	73.76	13.187	1.732
Hb preop-Hb postop1	No TXA	58	1.573	0.56	0.05
Hb preop-Hb postop1	TXA	137	1.176	0.15	0.02

**Table 2:** Correlation between TXA use and hemoglobin levels and total transfusion.

Correlations		Total transfusion	TXA	D5 PO Hgb	Pre-op Hgb	D1 PO Hgb
Total transfusion	Pearson correlation	1	−0.158^*∗*^	−0.200^*∗∗*^	−0.599^*∗∗*^	−0.450^*∗∗*^
	Sig. (2-tailed)		0.027	0.005	0.000	0.000
	*N*	195	195	194	195	195

TXA	Pearson correlation	−0.158^*∗*^	1	0.025	0.023	0.160^*∗*^
	Sig. (2-tailed)	0.027		0.726	0.751	0.026
	*N*	195	195	194	195	195

D5 PO Hgb	Pearson correlation	−0.200^*∗∗*^	0.025	1	0.277^*∗∗*^	0.436^*∗∗*^
	Sig. (2-tailed)	0.005	0.726		0.000	0.000
	*N*	194	194	194	194	194

Pre-op Hgb	Pearson correlation	−0.599^*∗∗*^	0.023	0.277^*∗∗*^	1	0.474^*∗∗*^
	Sig. (2-tailed)	0.000	0.751	0.000		0.000
	*N*	195	195	194	195	195

D1 PO Hgb	Pearson correlation	−0.450^*∗∗*^	0.160^*∗*^	0.436^*∗∗*^	0.474^*∗∗*^	1
	Sig. (2-tailed)	0.000	0.026	0.000	0.000	
	*N*	195	195	194	195	195

^*∗*^Correlation is significant at the 0.05 level (2-tailed). ^*∗∗*^Correlation is significant at the 0.01 level (2-tailed). Pre-op = preoperative, Hgb = hemoglobin, D = day, PO = postoperative.

**Table 3:** *t*-test for the TXA group and the control group.

Independent samples test										
		Levene's test for equality of variances		*t*-test for equality of means						
		*F*	Sig.	*T*	D*f*	Sig. (2-tailed)	Mean difference	Std. error difference	95% confidence interval of the difference	
									Lower	Upper
Pre-op Hgb	Equal variances assumed	4.680	0.032	−0.317	193	0.751	−0.0913	0.2879	−0.6591	0.4765
	Equal variances not assumed			−0.350	136.438	0.727	−0.0913	0.2605	−0.6064	0.4238

D1 PO Hgb	Equal variances assumed	0.000	0.988	−2.247	193	0.026	−0.4884	0.2174	−0.9172	−0.0597
	Equal variances not assumed			−2.253	108.127	0.026	−0.4884	0.2167	−0.9180	−0.0588

D5 PO Hgb	Equal variances assumed	0.486	0.487	−0.351	192	0.726	−0.0580	0.1655	−0.3844	0.2684
	Equal variances not assumed			−0.344	100.365	0.732	−0.0580	0.1689	−0.3930	0.2770

Total transfusion	Equal variances assumed	1.450	0.230	2.225	193	0.027	0.480	0.216	0.055	0.906
	Equal variances not assumed			2.390	127.083	0.018	0.480	0.201	0.083	0.878

Age	Equal variances assumed	1.707	0.193	−1.367	193	0.173	−3.335	2.440	−8.149	1.478
	Equal variances not assumed			−1.495	132.919	0.137	−3.335	2.232	−7.749	1.079

Pre-op = preoperative, Hgb = hemoglobin, *D* = day, PO = postoperative.

## Data Availability

The data used to support the findings of this study are available from the corresponding author upon request.
